# A brief cognitive-behavioural group therapy programme for the treatment of depression in adolescent outpatients: a pilot study

**DOI:** 10.1186/1753-2000-8-9

**Published:** 2014-03-22

**Authors:** Joana Straub, Nina Sproeber, Paul L Plener, Joerg M Fegert, Martina Bonenberger, Michael G Koelch

**Affiliations:** 1 Department of Child and Adolescent Psychiatry and Psychotherapy, University Hospital, Ulm, Germany; 2 Department of Child and Adolescent Psychiatry, Psychotherapy and Psychosomatics, Vivantes Hospitals, Berlin, Germany

## Abstract

**Background:**

The goal of this pilot study was to examine the feasibility and clinical outcomes of a brief (6-session) group therapy programme in adolescent outpatients with depression. The programme had previously been assessed in in-patients, with positive results.

**Methods:**

A total of 15 outpatients aged 13 to 18 years took part in the programme between October 2010 and May 2011, in 3 separate groups of 4–6 participants each. The outcomes measured were feasibility of the programme, as assessed by attendance rate, user feedback, fidelity of implementation, and response to treatment, as assessed by pre- and post-intervention measurement of depressive symptoms, quality of life, and suicidal ideation.

**Results:**

The programme demonstrated good feasibility, with a mean attendance rate of 5.33 out of 6 sessions, a mean rating by participants on overall satisfaction with the programme of 7.21 out of 10 (*SD* = 1.89), and a 93% concurrence between the contents of the sessions and the contents of the treatment manual. Compared to baseline scores, depressive symptoms at follow-up test were significantly reduced, as assessed by the Children’s Depression Rating Scale Revised (*F*(1, 12) = 11.76, *p* < .01) and the Beck Depression Inventory Revision (*F*(1, 32) = 11.19, *p* < .01); quality of life improved, as assessed by the Inventory of Quality of Life (*F*(1, 31) = 5.27, *p* < .05); and suicidal ideation was reduced. No significant changes were seen on the measures of the Parent Rating Scale for Depression and the Clinical Global Impression scale.

**Conclusions:**

Based on the results of this pilot study, it is feasible to further assess this brief outpatient treatment programme in a randomized controlled trial without further modifications.

## Background

The rate of depressive disorders in German adolescents aged 11–17 years is reported to be 4.7% for males and 9.7% for females [1]. Up to two-thirds of depressed adolescents suffer from co-morbid disorders [2], and depression is often associated with poor health behaviours and social challenges as well as with an elevated risk for suicide [3]. Suicide is the second most common cause of death for adolescents in Europe [4]. Given the nature and associated risks of depression, the Global Burden of Disease Study of the World Health Organization [5] has identified it as one of the most prevalent and debilitating disorders worldwide.

Several authors have looked at factors that might affect the effectiveness of therapies for children and adolescents with depression. In a meta-analysis of cognitive behavioural therapy (CBT) treatments, Weisz et al. [6] found a mean effect size (comparable to Cohen’s d) of .34. Group therapy was found to have several advantages over individual therapy, as follows: the group process can positively affect recovery, group members can learn from each other and give each other feedback, there is less stigmatisation, and the process may be more economical. A disadvantage, however, is that individuals with social anxiety or introversion may not receive as much attention as other participants [7]. In a recent meta-regression analysis looking at treatments for depression in adults, Cuijpers et al. [8] found that more sessions per week led to a larger effect size while every additional week of therapy reduced the effect size, which suggests there may be advantages to interventions that are short and intensive. Goodyer et al. [9] found that about one-fifth of depressed adolescents responded to a brief initial intervention (mean of 3 sessions). Brief therapies have the advantage that they allow for faster access to treatment and can be offered as a first-line treatment, reserving longer therapies for individuals who fail to respond.

There are currently six cognitive behavioural therapy programmes available in Germany to depressed adolescents: three for the prevention of depression and three for treatment [10-15]. Of the treatment programmes, one is the German version of the Adolescent Coping With Depression Course (CWD-A), which consists of ten 2-hour sessions [12]; one provides individual treatment sessions [11]; and one is primarily for the treatment of performance problems that may affect symptoms of depression [14]. However, none of these options combines the potential advantages described above of an intervention that uses CBT, is brief, and is delivered via group therapy. This article describes a brief (6-session) manualised programme that was developed to fill this gap. The programme is titled “**M**anualised **I**ntervention to **C**ope with depressive symptoms, **H**elp strengthen resources, and **I**mprove emotion regulation”, or MICHI, which is Japanese for “The Way” [16].

For safety reasons, as depression increases the risk for suicidal behaviour, the MICHI programme was first evaluated in in-patients [17]. The results of that initial pilot study, which was conducted in 9 adolescents (mean age = 16.33 years), showed good compliance in terms of the mean number of sessions attended (4.33 out of 5), positive user feedback on the programme content and the group leaders, improvement in symptoms of depression (CDRS-R: *z* = −2.66; *p* = .008), and reduction of suicidal ideation. Based on participants’ feedback, we changed several aspects of the programme, including reordering of the therapeutic contents, deleting some content such as relaxation techniques, and adding an extra session.

### Objectives of the pilot study

Before evaluating MICHI in a randomized controlled trial, we chose to conduct a second pilot study, this time in out-patients. Overall reasons for conducting pilot studies are to test the feasibility of a process, and to obtain preliminary data on the response to treatment [18]. Feasibility was here defined through three measures. The first was attendance, since as depression goes hand in hand with a lowered level of psychosocial functioning, participants might be expected to miss some sessions. A participation rate of five out of six sessions (79%) was defined as acceptable [19]. The second measure was user acceptance, for which we designed a questionnaire that asked participants how favourably they viewed various elements of the programme. The third feasibility measure was fidelity of implementation; i.e., how closely the psychologists who conducted the sessions were adhering to the treatment protocol defined in the MICHI treatment manual. Fidelity rates for manualised treatments typically range between 80% and 94% [20-23], so we defined anything within this range as being acceptable. With respect to response to treatment, efficacy was assessed by administering diagnostic tests of depression pre- post-intervention and follow-up and looking to see if scores were reduced following treatment. Safety was assessed through pre- post-intervention and follow-up measurement of suicidal ideation, which is a frequent symptom of depression. Since one of the goals of MICHI is to educate patients on how to deal with acute crises and prevent suicidal behaviour, we hypothesized that suicidal ideation would be reduced after participation.

## Methods

### Population

The pilot study was carried out in 3 separate groups of 4 to 6 participants each, with the first group starting in October 2010, the second in February 2011, and the third in March 2011. Recruitment was done by asking clinicians of local outpatient child and adolescent psychiatry and other local outpatient mental health institutions to refer any suitable patients to attend an information meeting held by the MICHI group leaders. Individuals who expressed interest following this meeting were scheduled for a screening visit, and those found eligible were enrolled in the next available group. Participants had to be aged between 13 and 18 years and to have an IQ of at least 80, a raw summary score on the Children’s Depression Rating Scale Revised (CDRS-R) [24] of at least 36 [25], and a diagnosis of a mild, moderate, or severe major depressive episode according to ICD-10 criteria [26]. In order for the sample to be representative of a naturalistic population, it was decided to not exclude difficult-to-treat patients [27]. Thus, co-morbid diagnoses were permitted as long as symptoms of depression were the main cause of the patient seeking medical support; and antidepressant medication was permitted provided it was stable for at least 5 weeks prior to study start and during the study. Individuals with a diagnosis of bipolar disorder, schizophrenia, or severe substance abuse were excluded, as these disorders require a different form of treatment and could be disruptive to the group process. Finally, participants and their parents/guardians had to agree to not to initiate any new drug treatment or new form of psychotherapy while participating in MICHI.

The study was approved by the IRB of the University of Ulm, Germany, and informed consent was provided by participants and their parents or guardians.

### Study design

As described in its treatment manual [16], MICHI is a CBT treatment that combines a number of therapeutic components widely acknowledged to represent the standard of care [28], [29]. All sessions included (1) psycho-education, (2) cognitive restructuring with the aim of reducing rumination [30], (3) behavioural activation, (4) resource activation, (5) enhancement of self-esteem, (6) problem-solving skills, (7) emotion regulation, (8) management of acute crises, and (9) prevention of relapse.

The programme included 5 weekly visits of 75 to 90 minutes in length, plus a “booster” session held 5.5 weeks after the last regular visit. An overview of the content and activities of the sessions is shown in Table 1. Each session included a review of what had been covered the previous week, provision of new information, therapist-assisted practice, homework for the coming week, assessment of mood using the Beck Depression Inventory–Revision (BDI-II) questionnaire [31], and the opportunity to talk to the therapist individually after the session in cases of acute crisis. Item 9 of the BDI-II, which asks about suicidal behaviour, was checked at every session, and if a participant reported current suicidal ideation, the programme supervisor was to be consulted in order to conduct a risk assessment and to decide if hospitalization was necessary. For Session 5, each participant was asked to bring along a “person of trust”, either a family member or a friend, who would attend the session and be trained on how to support the patient to help prevent relapse as well as on stepwise problem solving.

**Table 1:** Contents of MICHI sessions

**Session number**	**Contents**	**Exercises**
**Session 1**	• Get to know each other	• Postcards with different emotional motifs were displayed, and participants were encouraged to choose one that represented their depression best
	• Psychoeducation about symptoms of depression	
		• Related to the postcards, each participant was asked to tell his/her symptoms, and group leaders highlighted typical symptoms of depression
		• Homework: participants were asked to
		- Think about individual possible causes for their depressive symptoms
		- Conduct a positive activity each day and to evaluate how it affects their mood
		- Bring an object to the next session that represents something they are good at/proud of (e.g., football)
**Session 2**	• Resource activation	• Participants showed the object they brought that represented something they were good at/ proud of
	• Input about relationship between thoughts, behaviour, and feelings	• Participants were asked to name additional strengths and resources
	• Psychoeducation a bout causations of depression	• Psychoeducation about causation of depression (e.g., neurotransmitters, genetics, stressors)
		• Homework: participants were asked to
		- Focus on a positive and negative moment each day and to note down their behaviour, thoughts, and feelings in each moment
		- Note down compliments they receive or positive moments that happen to them in a diary
**Session 3**	• Enhancement of self-esteem	• Participants threw each other a ball, and each time they caught the ball they were asked to name a certain individual strength
	• Increase of behavioural activation	
		• Participants were invited to write each other compliments in their diaries
	• Psychoeducation about dysfunctional cognitions	• Input about the importance of positive self-esteem
		• Input about how errors in reasoning, e.g., dichotomous thinking, negatively influences how one feels
**Session 4**	• Repetition of contents	• Participants listened to an audiotaped interview with a depressed girl who talked about her symptoms, and were asked to give her advice about what she could do to feel better, taking into account what they learned in MICHI so far
	• Management of acute crises	
	• Emotion regulation	
		• Discussion and input about how to behave in case of crises (e.g., suicidal ideation)
	• Restructuring of dysfunctional cognitions	
		• Identification of helpful skills
		• Input about how to recognize negative thoughts and how to turn them into positive ones
**Session 5**	• Problem-solving skills prevention of relapse	• Participants learned how to solve problems in a theoretical stepwise manner; afterwards, they watched a video about a girl who is being bullied, and were asked how they would solve a problem like the one of the protagonist, taking into account the stepwise manner of problem-solving they learned before
		• Participants brought a person of trust
		• Conversation about how persons of trust can support participants in the future to prevent relapse
**Booster session 6**	• Recapitulation of contents of MICHI	• Contents of MICHI were repeated by means of a quiz
		• Each participant was asked to recapitulate his/her mood since the last session of MICHI
		• In case they found themselves in a depressed mood, they were asked whether they were able to apply elements of MICHI to prevent themselves from relapse
		• Participants were given a written case report of a depressed boy and were asked to advise him what he could do to feel better with reference to the contents learned in MICHI

The sessions were conducted according to the detailed instructions provided in the MICHI treatment manual, and were led by two of the manual’s co-authors (JS and MB) who are psychologists with master’s degrees and prior group CBT experience with adolescents. Both leaders were equally involved, and had predefined tasks to ensure that the same intervention was provided at all three MICHI groups. The group leaders were supervised by the manual’s main author and licensed psychotherapist (NS).

**Figure 1:**
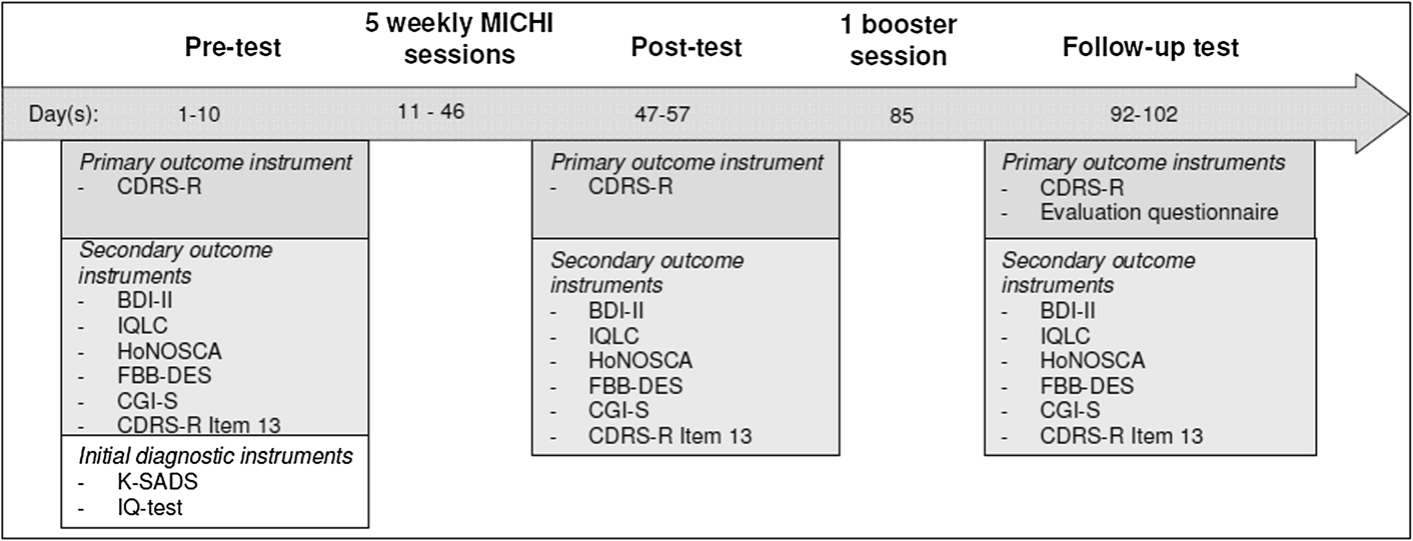
Study process and diagnostic instruments of MICHI.

The study design is presented in Figure 1. Assessments were carried out prior to Session 1 (pre-intervention), following Session 5 (post-intervention), and between 1 and 2.5 weeks after the booster session (follow-up). Each of the 3 assessment periods spanned 10 days, to allow for evaluation of all participants in the group.

### Diagnostic instruments

Screening measures included administration of the Kiddie Schedule for Affective Disorders and Schizophrenia, Present and Lifetime Version (K-SADS-PL) [32] to assess psychiatric disorders, and one of the following tests to assess intelligence: either the Wechsler Intelligence Scale for Children–Fourth Edition (WISC IV) [33], the Wechsler Adult Intelligence Scale (WAIS) [34], or the testing system for educational counselling (PSB) [35].

The primary outcomes for assessment of MICHI were: 1) feasibility of the programme, as assessed by attendance rate, user acceptance, and fidelity of implementation, and 2) preliminary data on the response to treatment assessed by the change in depressive symptoms, as measured by the CDRS-R total score. Attendance was defined as the percentage of sessions for which participants showed up. User acceptance was determined by having participants anonymously complete an ad hoc evaluation form containing 21 statements (α = .92 in the present sample) that were rated on a scale of 1 (not true) to 5 (true) plus one global question regarding overall satisfaction with the programme, rated on a scale of 1 (very poor) to 10 (very good). To determine fidelity of implementation, all sessions were videotaped, and 25% were randomly selected and viewed by an independent clinician to see if the required elements in the MICHI treatment manual were being implemented. A 57-item checklist was employed in the rating, with each item scored as either present or absent. The CDRS-R is a semi-structured clinician-rated interview, consisting of 17 questions, which asks about symptoms of depression over the last 2 weeks [24]. A score of 36 or greater indicates evidence of clinically relevant symptoms of depression [25]. The interview has shown a high internal consistency in previous studies with larger sample sizes (α = .90) [36] and α = .79 in the present sample). The German version of the CDRS-R was used in this study [37].The independent evaluators who performed the interview were blinded to the time points of measurement.

The secondary outcomes were changes in depression as measured by other instruments, changes in suicidal ideation, and changes in other measures of mental health. Two instruments were used for assessing depression over the last 2 weeks, one administered to the patient and the other to the parent/guardian: the BDI-II [31], which consists of 21 items (*α* = .95 in the present sample), and the Parent Rating Scale for Depression (FBB-DES), which is part of the diagnostic system for mental disorders in childhood and adolescence (DISYPS-II) [38] and is based on the international classification systems ICD-10 and DSM-IV (29 items; α = .89 in the present sample). Suicidal ideation was assessed by Item 13 of the CDRS-R (asking for suicidal ideations and suicide attempts). The question is answered using a 7-item scale ranging from “none to mild” (never thought about suicide or thought about it very seldom) to “moderate to severe” (thought about or attempted suicide within the last month). Other instruments used were the Inventory for the Assessment of Quality of Life in Children and Adolescents (IQLC) [39], which consists of 7 items (α = .59 in the present sample) and assesses quality of life in the past week; the Clinical Global Impression (CGI) [40], which consists of 1 item and assesses severity of symptoms over the past week; and the Health of the Nation Outcome Scales for Children and Adolescents (HoNOSCA) [41], which consists of 13 items (α = .74 in the present sample) and assesses psychosocial strain over the past month. The time points at which each instrument was administered are shown in Figure 1.

### Statistical methods

All analyses were conducted using mixed effects repeated measures analysis (multi-level) for longitudinal data with an autoregressive covariance structure (AR1) and maximum likelihood estimation which is a contemporary method for handling missing data [42]. Comparisons between small samples were done by means of the Mann–Whitney test. Statistical analyses were performed using PASW statistics 18. For testing hypotheses, the significance level was set a priori at a two-tailed type I error rate of .05. To evaluate the impact of treatment, effect sizes (*ω*^*2*^ omega squared) were calculated for ANOVA with repeated measurements. Interpretation of the effect size *ω*^*2*^ is as follows: .01 *≤ ω*^*2*^ *<* .06 – small effect; .06 *≤ ω*^*2*^ *<* .14 – moderate effect; *ω*^*2*^ ≥ .14 – large effect [43].

## Results

### Patient disposition and characteristics

**Figure 2:**
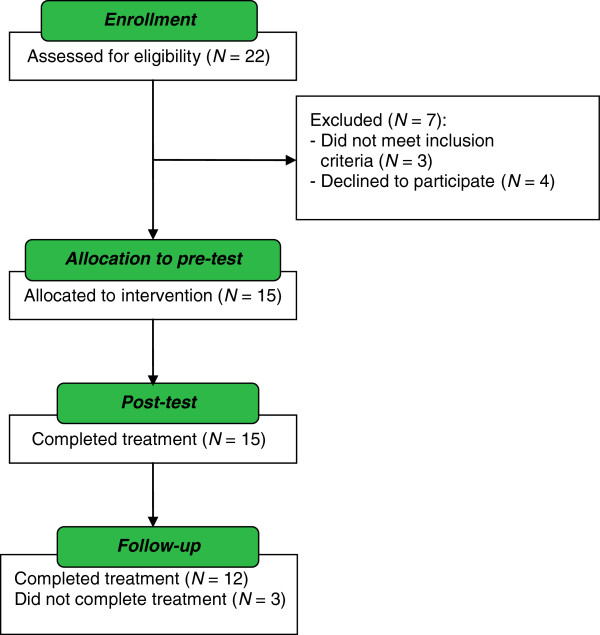
Flowchart of participation in MICHI.

Patient disposition is shown in Figure 2. Of 22 adolescent outpatients who were referred for screening, 3 were screening failures (reasons: IQ < 80, CDRS-R score < 36, and patient started medication during the diagnostic period), and 4 declined to participate; 3 by their own choice and 1 where permission was refused by the mother. The pre-intervention CDRS-R scores of the 4 individuals who declined participation did not differ significantly from those of the individuals who did take part (*U* = 25, *z* = −.50, *p* = .62). Of the 15 outpatients who were enrolled in the study, 12 (80.0%) attended all visits, while 3 (20.0%) dropped out before the follow-up visit. The reasons for non-completion were starting individual psychotherapy for treatment of co-morbid social phobia, starting inpatient treatment as the participant was no longer able to comply with Germany’s policy regarding compulsory school attendance, and starting on antidepressant medication prior to the follow-up assessment. The pre-intervention CDRS-R scores of the non-completers did not differ from those of the completers (*U* = 14.5, *z* = −.51, *p* = .61); however, 2 of these individuals differed from the others with respect to co-morbid diagnosis (social phobia). After MICHI, nine patients had further regular visits to a psychiatric clinic or practice, meeting with a psychiatrist/psychologist for approximately 30 minutes once every 1–3 months. These appointments helped to stabilize or support them. Two were referred for further weekly psychotherapy.

Demographic and diagnostic data of the patients who took part in the study are provided in Table 2. Mean age was 16.42 (SD = 1.43) years (range: 13.1 to 17.9 years), and 11 (73.3%) were female. Four (26.6%) had previously received psychological treatment, but none were receiving it currently or had received it within the last 12 months.

**Table 2:** Demographics and pre-post-follow-up test sum scores of participants

**Participant**	**Sex**	**IQ**	**Depression diagnoses**	**Co-morbid diagnoses**	**Medication**	**Diagnostic instrument**	**Pre-test**	**Post-test**	**Follow-up test**
1	F	97	F32.2	-	-	CDRS-R	65	55	36
						BDI-II	-	17	15
2	M	102	F32.1	-	-	CDRS-R	48	43	43
						BDI-II	9	9	9
3	F	95	F32.1	-	-	CDRS-R	57	53	49
						BDI-II	28	28	15
4	F	94	F32.1	-	-	CDRS-R	52	45	49
						BDI-II	33	30	30
5	F	94	F32.2	-	-	CDRS-R	72	56	34
						BDI-II	39	38	20
6	F	92	F32.1	-	-	CDRS-R	50	63	58
						BDI-II	39	30	28
7	M	100	F32.2	-	-	CDRS-R	61	60	61
						BDI-II	17	26	20
8	F	102	F32.0	-	-	CDRS-R	40	30	30
						BDI-II	8	3	4
9	F	100	F32.2	F40.1	-	CDRS-R	63	49	Drop out
						BDI-II	44	47	
10	F	114	F32.0	-	-	CDRS-R	36	37	23
						BDI-II	3	1	0
11	F	111	F33.0	-	-	CDRS-R	45	27	18
						BDI-II	34	19	3
12	F	106	F32.0	F40.1	-	CDRS-R	45	31	30
						BDI-II	15	8	7
13	F	100	F32.1	-	-	CDRS-R	64	48	58
						BDI-II	23	20	23
14	M	111	F32.1	-	Fluoxetin	CDRS-R	60	32	Drop out
						BDI-II	42	6	
15	M	104	F32.1	F40.1	St. John’s wort	CDRS-R	49	60	Drop out
						BDI-II	39	28	

***Notes*** F32.0 = mild depressive disorder; F32.1 = moderate depressive disorder; F32.2 severe depressive disorder; F33.0 = recurrent mild depressive disorder (diagnoses following ICD-10); BDI-II = Beck depression inventory; CDRS-R = children’s depression rating scale revised, F = female; M = male.

### Programme feasibility

Nine (60%) participants attended all 6 sessions, 3 (20%) attended 5 sessions, 2 (13.3%) attended 4 sessions, and 1 (6.6%) attended 3 sessions. The overall attendance rate was 78.8%, with a mean of 5.33 out of 6 sessions attended. At Session 5, 11 participants (73.3%) brought a supportive person as requested, 2 (13.3%) came alone, and 2 (13.3%) did not show up. Of those who brought somebody, 5 came with their best friend, 3 with their mother, 2 with a sibling, and 1 with a social worker who worked with her family.

The results of the user feedback questionnaire are presented in Table 3. The mean ratings ranged from 2.29 to 4.14 out of 5. The highest ratings (≥4.0) were seen for the statements regarding whether participants liked being in the group with other adolescents, whether they felt the programme would be helpful for others, and whether they felt comfortable with and understood by the group leaders. The lowest ratings (<3.0) were seen for the statements regarding whether participants felt that what they had learned in the programme could be successfully applied to their daily life, family life, leisure time, and school; whether they felt that family members and friends could help them with problems in future; and whether the inclusion of family and friends had been helpful. The mean score for the global question on overall satisfaction with the program was 7.21 (*SD* = 1.89) out of 10.

**Table 3:** Evaluation questionnaire for the assessment of acceptance

**Item**	**Mean rating**
I like learning with other adolescents	4.14
I expect the training to be helpful to other adolescents as well	4.07
I felt comfortable with the trainers	4.00
I felt well understood by the trainers	4.00
Advice of the trainers was helpful to me	3.93
I felt comfortable within the group	3.59
The exercises in the training were helpful	3.50
It is helpful to learn with other adolescents	3.43
I expect that the things I learned will help me in the future	3.29
The amount of homework was helpful	3.14
The homework in general was helpful	3.14
I liked the inclusion of family and friends	3.14
I would participate again in a training like MICHI	3.14
I expect to be able to implement the things I learned in the future	3.07
The transfer of the things learned in the homework to daily life was successful	2.93
The things learned were helpful in my leisure time	2.86
I expect that family and friends can help me with my problems in the future	2.79
The inclusion of family and friends was helpful	2.64
The things learned were helpful to me in school/vocational training	2.57
The things learned were helpful in my family life	2.29

***Notes*** Evaluation questionnaire of MICHI (answers range from 1 to 5; the higher the number, the stronger the agreement).

With respect to fidelity to the MICHI treatment manual, based on the findings of the independent clinician who rated videotapes of randomly selected sessions, there was a 93% concurrence between the manualised treatment and what was delivered.

### Response to treatment

**Figure 3:**
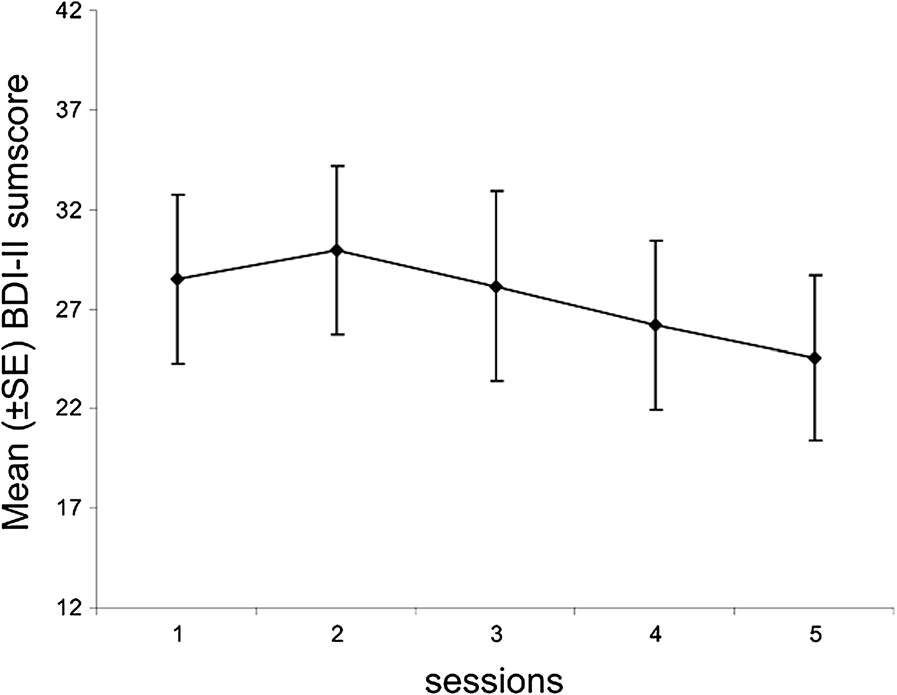
BDI-II mean sum score of participants per MICHI session.

The total CDRS-R scores decreased significantly from pre-intervention to follow-up assessment (*F* (1, 12) = 11.76, *p* < .01), and 5 (42%) of the 12 participants who completed the follow-up assessment no longer met the criteria for clinically significant depression (CDRS-R score < 36). Scores also decreased significantly for the BDI-II (*F* (1, 32) = 11.19, *p* < .01) and the HoNOSCA (*F* (1, 37) = 4.54, *p* < .05) and increased significantly for the IQLC (*F* (1, 31) = 5.27, *p* < .05). No significant changes were seen on the FBB-DES and the CGI. See Table 4. Furthermore Figure 3 demonstrates the course of the BDI-II assessments per session. Results revealed a decline of symptom severity from session two onwards.

**Table 4:** Results of diagnostic instruments

	**M**	**SD**	**N**	**F**	**df**_M_	**df**_R_	**p**	**ω**^**2**^
***Primary outcome***								
**CDRS-R**								
Pre-test	53.80	12.24	15					
Post-test	45.93	12.11	15					
Follow-up	40.75	14.39	12	11.76	1	11.92	.005**	.11
***Secondary outcomes***								
**BDI-II**								
Pre-test	26.64	14.00	14					
Post-test	20.67	13.45	15					
Follow-up	14.50	9.98	12	11.19	1	31.97	.002**	.01
**IQLC**								
Pre-test	20.60	3.85	15					
Post-test	19.13	4.03	15					
Follow-up	18.42	4.19	12	5.27	1	30.57	.03*	.03
**HoNOSCA**								
Pre-test	13.60	5.88	15					
Post-test	13.14	6.02	14					
Follow-up	9.25	5.14	12	4.54	1	36.92	.04*	.04
**FBB-DES**								
Pre-test	21.93	14.18	15					
Post-test	23.31	12.30	13					
Follow-up	17.11	7.90	9	.94	1	27.02	.34	.01
**CGI-S**								
Pre-test	4.00	1.07	15					
Post-test	4.00	1.00	15					
Follow-up	3.33	1.56	12	2.11	1	14.37	.17	.01

***Notes*** * p < .05, ** p < .01; high mean values demonstrate a higher symptom severity; Explanation of abbreviations: *M* = mean; *SD =* standard deviation; *N* = number of participants; *df*_M_ = degrees of freedom for the effect of the model; *df*_R_ = degrees of freedom for the residuals of the model; effect sizes were analysed according to ANOVA with repeated measurements; *ω*^*2*^ = omega squared effect size; Interpretation of the effect size ω^2^: .01 ≤ ω^2^ < .06 – small effect; .06 ≤ ω^2^ < .15 – moderate effect; ω^2^ ≥ .15 – large effect.

With respect to suicidal ideation, pre-intervention, just 4 (26.7%) participants had a response of “none to mild” on Item 13 of the CDRS-R while 11 (73.3%) had a response of “moderate to severe”. Post Session 5, these numbers were 10 (67%) and 5 (33%), respectively; and at follow-up, they had further improved to 10 (80.0%) and 2 (20.0%), respectively. The change from pre-intervention to follow-up was statistically significant (*F* (1, 32.81) = 4.25, *p* < .05).

## Discussion

The aim of this pilot study was to assess a brief cognitive behavioural group therapy programme in adolescent out-patients suffering from depression. The results showed good feasibility and significant clinical improvements. The adolescents in this programme participated on a regular basis and rarely missed sessions, despite the low level of psychosocial functioning usually associated with a major depressive disorder.

With regard to acceptance, the most positive evaluations were given for being with other adolescents and feeling comfortable in the group. One reason for this could be that compared to their healthy peers, depressed adolescents have more problems with social relationships, fewer contacts with peers, and more likelihood of social rejection [44]. Individuals with depression often have social deficits that weaken their chances for social reinforcement [45]; thus, the opportunity for positive social experiences in a protected setting in which they feel comfortable would be highly important to them. However, considering the drop-outs more closely, it becomes obvious that two of three had a comorbid social phobia. As written in the introduction, the group setting might be overly stressful and less appropriate for them than the individual setting. Participants also reported that they felt understood by and comfortable with the group leaders, and rated their advice as helpful. The importance of a positive therapeutic alliance and its effect on therapeutic outcome has been demonstrated elsewhere [46], and could be a nonspecific factor that at least partly explains the improvement in symptoms.

The least positive evaluations were on whether participants found what they learned in the group to be helpful in their family lives. One reason for this could be that adolescents are at an age where they increasingly detach from their parents while friends become more important. It was notable that only 3 participants chose a parent as the “person of trust” to bring to Session 5 therefore this item might be appropriate for only some of the sample. A further reason could be that one session might not be enough to focus on all skills necessary. The addition of a cognitive behavioural family therapy component [47] might improve response rates.

Furthermore participants rated inclusion of family and friends little helpful. The reason for including the person of trust was to help prevent relapses; however, such assistance may not be necessary until much later. Therefore, assessing the value of this role shortly after completion of the program may not be useful, as the patient is unlikely to require such support at this time. The value of including and training a supportive friend or family member should be evaluated in a longitudinal design.

Adherence to the MICHI protocol was 93%, which is comparable to the range of 80% to 94% reported for other manualised studies [20-23]. The high fidelity of implementation may be attributable to the detailed instructions provided in the treatment manual.

Significant improvements were seen on measures of depression and the pre-post follow-up test comparison revealed a moderate effect size for the CDRS-R and a small effect size for the BDI-II. The remission rate of 42% was slightly lower than the rates of 45.2% and 56.0% seen by Ihle et al. [48,49], who used a similar pre-post design in their investigation of the German version of the “Adolescent Coping with Depression Course (CWD-A)”. It must be noted that the CDW-A programme, while brief, consists of ten 2-hour group sessions in comparison with only six 1.25-hour group sessions in MICHI. In any case, the remission rate in MICHI is within the range (39%–62%) seen with international studies for the evaluation of group treatments for depressed adolescents [22,23,50]. Due to the small number of participants in the present sample, the remission rate needs to be addressed in a larger sample as well.

No significant change was seen in the scores of the parent-rated FBB-DES. This could be explained by the fact that symptoms of depression, such as reduced self-esteem, feelings of depression, guilt and hopelessness, and suicidality, are difficult for parents to observe. This aspect was also reported by Weisz et al. [6], who noted that after therapy of depressed adolescents, the youth-completed reports revealed significant improvement while the parent-completed reports did not. Pre-post follow-up test comparisons in the IQLC and HoNOSCA revealed small effect sizes and the CGI scores improved slightly but not significantly, which again might be a factor of the small number of participants.

There was a significant reduction in the number of participants reporting suicidal ideation within the last 2 weeks, with only 20% still responding “moderate to severe” at follow-up. In a study of healthy European adolescents aged 14–16 years, Resch et al. [51] found that 17% of females and 8.3% of males reported having had suicidal thoughts within the last 2 weeks, as assessed by the Paykel Scale [52]. Thus, the percentage of adolescents with suicidal ideation seen in our sample follow-up-intervention is not much larger than that seen in a non-clinical population of the same age. This finding of a reduction of suicidal thoughts following outpatient treatment is important, as suicidality in adolescents is a major public health problem due to its frequency, likelihood for recurrence, increased risk for completed suicide, and health costs [53].

A comparison of the results of this pilot study with the earlier pilot study of MICHI in in-patients [17] revealed no difference in feasibility and a nearly identical improvement in pre-post CDRS-R scores. With respect to suicidal ideation, the out-patients showed a higher percentage of moderate to severe suicidal ideation pre-intervention than did the in-patients. Post-intervention, reduction of suicidal ideation was comparable between both samples.

Some limitations of this pilot study must be recognized. These include a small sample size; the absence of a control group, which allows for only weak conclusions about efficacy; a small cronbach’s alpha of the IQLC, which might be due to a small number of items and stronger changes in some items than others, and the inclusion of participants who were receiving medication for depression. We attempted to reduce the impact of medication by including only patients whose regimen was stable for five weeks prior to the start of and during therapy. Furthermore we included participants with co-morbid disorders that could influence results. It is possible that permitting patients to speak to a group leader individually following a session in case of an acute crisis could have influenced results; however, this opportunity was used only few times. This issue was discussed before the start of the programme, and was felt to be a necessary supplement for ethical reasons and because it reflects practical reality.

## Conclusions

The results of this pilot study revealed a reduction in depression and suicidal ideation and an improvement of quality of life in adolescent outpatients following participation in a brief manualised CBT group therapy programme. Overall, the findings support the feasibility of proceeding with an investigation of the MICHI programme using a larger sample size in a randomized controlled trial design. If the findings are borne out in a controlled trial, this approach could come to be considered a first-line treatment for adolescents with depression, reserving longer-lasting therapies or medical treatment only for those who fail to respond.

## Competing interests

All authors declare no conflict of interests.

## Authors’ contributions

PLP is PI in a study for Lundbeck. He has received research grants from the BMBF (German Ministries for Research and Education) and the BfArM (German Federal Institute for Drugs and Medical Devices) and the state foundation (Landesstiftung) Baden-Wuerttemberg. He has received travel grants from the DFG, DAAD and IACAPAP. He is not a shareholder in the pharmaceutical industry. JS managed the literature searches, acquisition of data, and analysis and interpretation of data, and was the primary author of the article. NS developed the MICHI training manual and designed the study. PLP aided in the literature search. MB conducted group therapy sessions and helped with acquisition of data. MGK supervised and coordinated the study. All authors contributed to the manuscript and approved the final version. JMF has received research funding in the last 5 years from EU, DFG, BMG, BMBF, BMFSFJ, several state ministries of social affairs, State Foundation BaWue, Volkswagen Foundation, European Academy, Gregorian University, Vatican, RAZ, CJD, Eli Lilly research foundation, Janssen-Cilag (J&J), Medice, Celltech/UCB. Furthermore, he has received travel grants, honoraria, and sponsoring for conferences and medical educational purposes from DFG, AACAP, NIMH/NIH, EU, the Vatican, Goethe Institute, Pro Helvetia, Astra, Aventis, Bayer, Bristol-MS, Celltech/ UCB, Janssen-Cilag (J&J), Lilly, Medice, Novartis, Pfizer, Ratiopharm, Sanofi-Synthelabo, Shire, VfA, Generikaverband, several universities and professional associations, and German federal and state ministries. MGK has received unrestricted grants from Eli Lilly International Foundation. He has received research grants from the BMFFSJ (German Ministries for Family Affairs, Senior Citizens, Women and Youth), the BMBF (German Ministries for Research and Education), the SchweizerBundesamt fuer Justiz, and BoehringerIngelheim. He has been a CI or PI for Eli Lilly, Astra Zeneca, and Janssen-Cilag, Lundbeck. He has received travel grants or payments for lectures from Janssen-Cilag, the University of Rostock, DGKJPP, UCB, EuropaeischeAkademie and from various non-profit organizations. He is not a shareholder in the pharmaceutical industry.
